# OR11-005 - Mast cells respond to pathogen signals with IL-1ß

**DOI:** 10.1186/1546-0096-11-S1-A194

**Published:** 2013-11-08

**Authors:** L Kraas, O Schmetzer, K Krause, E Latz, M Maurer

**Affiliations:** 1 Dermatology and Allergy, Charité Universitätsmedizin Berlin, Berlin, Germany; 2 Institute of Innate Immunity, University Hospitals Bonn, Bonn, Germany

## Introduction

Mast cells, key effector cells of allergic and innate immune responses, have recently been reported to be an important source of IL-1ß in patients with autoinflammatory conditions such as cryopyrin-associated-periodic-fever syndromes (CAPS). CAPS patients show IL-1beta-driven systemic inflammation together with non-histamine dependent urticarial rash, which are caused by activating mutations of the inflammasome, a multiprotein oligomer responsible for the initiation of inflammatory responses to pathogens.

## Objectives

To determine if mast cells can produce and release IL-1ß in response to pathogenic signals that target the inflammasomes NLRP3, NLRC4, or AIM2.

## Methods

Peritoneal mast cells (PMCs) were obtained through lavage from adult (>8 weeks) C57BL/6 mice and WBB6F1 Kit+/+ mice, purified via CD117+ bead selection (>96 % purity) and cultured for 7-14 days. 10^5^ cells/well were primed with LPS (100ng/ml) for 15 hrs. Then the PMCs were stimulated with 10 µM Nigericin (NLRP3), 5mM ATP (NLRP3), 100µM R837 (NLRP3) for 45 min or for 4 hours with 600 ng Flagellin (NLRC4) transfected with DOTAP or 200 ng polydAdT (AIM2) transfected with Lipofectamine. IL-1 beta production was measured in the supernatants by Elisa.

## Results

PMCs produced significant amounts (mean ± SEM) of IL-1ß upon stimulation with Nigericin (467 ± 41pg/ml), ATP (152 ± 88pg/ml), R837 (21 ± 2 pg/ml), Flagellin (245 ± 44pg/ml) and polydAdT (571 ± 194pg/ml) as compared to no stimuli (7,1 ± 0,8 pg/ml) only.

## Conclusion

We show that mouse mast cells incubated with inflammasome activators produce significant amounts of IL-1ß ex vivo. Our data suggest that inflammasome-driven mast cell activation and subsequent IL-1ß production and release may importantly contribute to innate immune responses to pathogens.

## Competing interests

None declared.

